# Study on predicting microsatellite instability in rectal cancer using T_2_ mapping combined with ADC value

**DOI:** 10.1186/s40644-026-00999-w

**Published:** 2026-01-30

**Authors:** XiaoXin Zhao, YueJiao Hou, HongZhou Ma

**Affiliations:** 1 Department of Magnetic Resonance Imaging, Heze Hospital of Shandong Provincial Hospital (Heze Municipal Hospital), Heze City, Shandong Province 274000 China; 2 Department of Radiologic Imaging, Heze Hospital of Shandong Provincial Hospital (Heze Municipal Hospital), No. 2888, Caozhou West Road, Heze City, Shandong Province 274000 China

**Keywords:** Rectal cancer, Microsatellite instability (MSI), T₂ mapping, Apparent diffusion coefficient (ADC), Magnetic resonance imaging (MRI)

## Abstract

**Objective:**

To investigate the feasibility of T₂ mapping combined with apparent diffusion coefficient (ADC) value for preoperatively predicting microsatellite instability (MSI) status in rectal cancer.

**Methods:**

This retrospective study included 152 patients with pathologically confirmed rectal cancer (40 MSI, 112 microsatellite stable [MSS]). All patients underwent MRI including T₂ mapping and diffusion-weighted imaging (DWI). Tumor T₂ and ADC values were measured and compared. A combined prediction model was constructed using multivariate logistic regression with SMOTE oversampling and L2 regularization. Diagnostic performance was evaluated using receiver operating characteristic (ROC) curve analysis, with model stability assessed via 10-fold cross-validation and Bootstrap resampling (1000 times).

**Results:**

The MSI group had a significantly lower T₂ value (92.18 ± 7.21 ms vs. 99.47 ± 7.85 ms, *p* < 0.001) and a higher ADC value (1.06 ± 0.18 vs. 0.91 ± 0.19 × 10⁻³ mm²/s, *p* < 0.001) compared to the MSS group. The AUC for predicting MSI status was 0.865 for T₂ value and 0.741 for ADC value. The combined model significantly improved the AUC to 0.915 (95% CI: 0.865–0.965), with a sensitivity of 82.5% and specificity of 89.3%. The model demonstrated excellent stability (Bootstrap mean AUC = 0.913).

**Conclusion:**

T₂ mapping combined with ADC value provides a reliable, non-invasive method for preoperative prediction of MSI status in rectal cancer. The combined model demonstrates higher diagnostic efficacy than either parameter alone and shows promising potential for clinical translation to support precision treatment decision-making in conjunction with histopathological assessment.

MSI (Microsatellite Instability) refers to a state of genomic instability caused by defects in the DNA mismatch repair (MMR) system, which prevents the correction of errors in the replication of small repetitive DNA sequences (microsatellites) during cell division [1, 2]. MSS (Microsatellite Stability) refers to a state of stable microsatellites. MSI tumors account for approximately 3–5% of all rectal cancers. Compared with MSS tumors, patients with stage II MSI-high (MSI-H) rectal cancer have a significantly lower recurrence risk and longer overall survival after surgery. Meanwhile, MSI-H/deficient MMR (dMMR) is the strongest biomarker for predicting the efficacy of immune checkpoint inhibitors [3, 4]. However, the current assessment of MSI status in rectal cancer relies on biopsy, and conventional imaging methods cannot directly evaluate the MSI status of rectal cancer [5, 6]. The purpose of this study was to investigate the feasibility of preoperatively predicting the MSI status of rectal cancer using T₂ mapping technique combined with ADC value.

## Materials and methods

This study complied with the Declaration of Helsinki and was approved by the Ethics Committee of Heze Hospital of Shandong Provincial Hospital. Since the study was a retrospective analysis with all patient data de-identified and no interventional procedures involved, it was exempt from requiring informed consent upon review by the Ethics Committee. and the approval number was 2025-KJKY022-074.

### General data

This retrospective analysis included 152 consecutive patients with pathologically confirmed rectal cancer who were treated at Heze Hospital of Shandong Provincial Hospital between January 2022 and June 2025.

Inclusion criteria were as follows: (1) All cases had complete pathological results, and MSI status was confirmed by immunohistochemistry (IHC) for mismatch repair proteins (MLH1, MSH2, MSH6, and PMS2) combined with MSI-PCR analysis of five microsatellite loci (BAT25, BAT26, D5S346, D2S123, and D17S250); (2) All patients were evaluated exclusively in the preoperative setting, and MRI examinations were performed prior to any treatment (including surgery, neoadjuvant chemotherapy, radiotherapy, or immunotherapy); (3) Image quality score ≥ 1 (see Sect. “Image Analysis” for definition).

Exclusion criteria included: (1) Severe image artifacts (e.g., motion or foreign-body artifacts) that impaired lesion delineation or quantitative measurement (image quality score = 0); (2) Receipt of intestinal anti-inflammatory treatment (e.g., 5-aminosalicylic acid) within 3 months before surgery; (3) Incomplete clinical or follow-up data.

Sample size estimation was performed based on a previously reported difference in T_2_ values between the MSI and MSS rectal cancers (9.06 ± 7.00 ms) [7]. With a significance level of α = 0.05 and a statistical power of 0.90, the minimum sample size for the MSI group was calculated as 35 using G*Power 3.1. To ensure statistical robustness, a total of 40 MSI patients were ultimately included in this study.

### Equipment and methods

MRI examinations were performed using 3.0T superconducting MRI scanner (Philips Elition, Philips Healthcare, the Netherlands, with an 18-channel phased-array body coil; and Siemens Prisma, Siemens Healthineers, Germany, with a 32-channel phased-array body coil). All patients were examined in the supine position. Prior to MRI examination, patients were instructed to empty the rectum. No formal bowel preparation, enema, fasting, or antispasmodic agents were administered.

High-resolution T2-weighted imaging (T2WI) was acquired according to standard rectal MRI protocols. Oblique axial T2WI was obtained perpendicular to the long axis of the tumor for optimal local assessment. Sagittal T2WI was routinely acquired for anatomical reference and evaluation of tumor extent, while coronal images were obtained when necessary to further delineate lesion morphology. The detailed scanning parameters were as follows: (1) Oblique axial high-resolution T2WI: repetition time (TR) = 2500 ± 100 ms, echo time (TE) = 100 ± 5 ms, field of view (FOV) = 20–22 cm × 20–22 cm, matrix = 320 × 240, slice thickness = 3 mm, slice gap = 0.4 mm; fat suppression method: chemical shift suppression. (2) Diffusion-weighted imaging (DWI): TR = 4360 ± 200 ms, TE = 77 ± 3 ms, FOV = 30 cm × 22 cm, matrix = 168 × 168, slice thickness = 3 mm, slice gap = 0.4 mm, b-values = 50 and 800 s/mm², diffusion directions = 15. (3) T₂ mapping: Using the mDIXON QUANT sequence; TR = 1700 ± 50 ms, TE = 13, 26, 39, 52, 65, 78 ms, flip angle (FA) = 90°, echo train length (ETL) = 22, FOV = 38 cm × 38 cm, number of excitations (NEX) = 1, slice thickness = 3.5 mm, slice gap = 2.5 mm.

T2 mapping was included to enable quantitative and objective assessment of tissue microstructural properties. Compared with conventional T2-weighted signal intensity, T2 mapping provides reproducible quantitative measurements and reduces subjectivity related to visual interpretation, thereby improving interobserver consistency.

To evaluate the consistency of measurement results between different MRI systems, 20 patients were randomly selected for inter-scanner comparison. T2 and ADC values were independently measured by the same radiologist on images acquired from both scanners, and Bland-Altman analysis was performed to evaluate measurement agreement.

### Image analysis

Three senior radiologists with more than 8 years of experience in abdominal MRI diagnosis independently measured the T₂ values and ADC values using Philips Intellispace Portal (Version 11.1) or Siemens Syngo VIA workstation in a blinded manner (unaware of patients’ clinical/pathological data).

Criteria for drawing the region of interest (ROI): (1) Slice selection: 3 slices (the maximum cross-section of the lesion + 1 adjacent slice above and below); (2) ROI range: Covering the entire solid component of the tumor, avoiding visible necrotic areas (T2WI signal ≥ 2 times the muscle signal), hemorrhagic areas, mucus, and the muscular layer of the intestinal wall; (3) ROI area: 15–30 mm² per slice; the final value was the average of the 3 slices [8].

Consistency verification: If the measurement difference among the 3 radiologists exceeded 10%, a consensus was reached through joint image review; otherwise, the average value was taken. The coefficient of variation (CV, ≤ 15% considered acceptable) was used to evaluate intra-group consistency, and the intraclass correlation coefficient (ICC, ≥ 0.90 considered excellent) was used to assess inter-observer consistency.

Image quality scoring: 0 points: Severe artifacts (tumor boundary unidentifiable, unable to draw ROI); 1 point: Mild artifacts (tumor boundary clear, no impact on ROI drawing); 2 points: No artifacts (uniform tumor signal, clear structure).

### Statistical analysis

SPSS 26.0 and R 4.3.1 software were used for statistical analysis. (1) Grouping criteria: MSS group: All 4 IHC markers positive or only 1 marker missing [MSI-low (MSI-L)]; MSI group: ≥ 2 IHC markers missing, confirmed by MSI-PCR. (2) Normality test: Kolmogorov-Smirnov test; Homogeneity of variance test: Levene test. (3) Univariate analysis: Independent samples t-test (comparison of T2/ADC values between groups); one-way analysis of variance (ANOVA) (comparison of different clinical stages). (4) Multivariate analysis: Binary logistic regression combined with SMOTE oversampling (to address class imbalance, oversampling ratio = 1:1, k-nearest neighbors = 5) and L2 regularization (to avoid overfitting, λ = 0.01) was used, and confounding factors (BMI, tumor location, image quality score) were included in the model. (5) Model validation: 10-fold cross-validation (Stratified by MSI status) and Bootstrap resampling (1000 times) were used to evaluate stability; Delong test was applied to compare the AUC of single/combined parameters.

## Results

### Baseline characteristics of patients

**Fig. 1:**
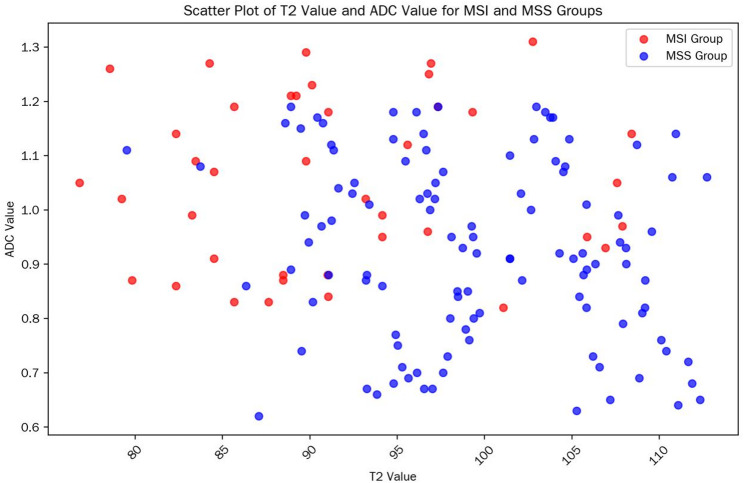
Scatter plot of T2/ADC values in each group; data points in the MSI group are concentrated in the “low T2 value-high ADC value” region

A total of 152 patients with pathologically confirmed rectal cancer were included in this study and divided into two groups according to microsatellite status: 40 cases in the MSI group (accounting for 26.3%, with 12/18/10 cases in clinical stage I/II/III, respectively) and 112 cases in the MSS group (accounting for 73.7%, with 28/50/34 cases in clinical stage I/II/III, respectively). There were no statistically significant differences in baseline characteristics such as age, gender, BMI, tumor location, clinical stage, and image quality score between the two groups (all *p* > 0.05), indicating comparability; only the T₂ value and ADC value showed significant differences between the two groups (*p* < 0.001). The statistical data are shown in Table 1. The scatter plot of T2/ADC values in each group is shown in Fig. 1.

**Table 1:** Baseline characteristics of patients and inter-group comparative analysis

Parameter	MSI group (*n* = 40)	MSS group (*n* = 112)	Test statistic	*p*-value
Age (years)	62.5 ± 10.3	63.8 ± 9.6	t = -0.71	0.478
Gender (male/female)	18/22	60/52	χ² = 0.12	0.728
BMI (kg/m²)	24.1 ± 2.3	23.8 ± 2.5	t = 0.65	0.516
Tumor location (low/middle/high)	15/17/8	42/50/20	χ² = 0.58	0.748
T₂ value (ms)	92.18 ± 7.21	99.47 ± 7.85	t = -5.89	< 0.001
ADC value (×10⁻³ mm²/s)	1.06 ± 0.18	0.91 ± 0.19	t = 4.78	< 0.001
Clinical stage (I/II/III)	12/18/10	28/50/34	χ² = 0.35	0.84

Note: Low rectal cancer was defined as ≤ 5 cm from the anal verge, middle rectal cancer as 5–10 cm, and high rectal cancer as > 10 cm; *p* ≤ 0.05 was considered statistically significant.

The consistency of measurement results among three radiologists was evaluated using the intraclass correlation coefficient (ICC). The results showed that the measurements of T2 values and ADC values both exhibited excellent inter-rater consistency (ICC_T2 = 0.94, 95% CI: 0.91–0.96; ICC_ADC = 0.92, 95% CI: 0.88–0.95).

The consistency between IHC and MSI-PCR results was excellent: Kappa value = 0.92 (95% confidence interval [CI]: 0.85–0.99), with 145 consistent cases (accounting for 95.4%), including 38 consistent cases in the MSI group (95.0%) and 107 consistent cases in the MSS group (95.5%), which verified the reliability of MSI status determination. In terms of image quality, 110 cases (72.4%) scored 2 points (no artifacts, uniform tumor signal, clear structure), and 42 cases (27.6%) scored 1 point (mild artifacts, no impact on ROI drawing); no cases scored 0 points (severe artifacts), which met the measurement requirements.

### Results of univariate analysis

T₂ value: The T₂ value in the MSI group was significantly lower than that in the MSS group (92.18 ± 7.21 ms vs. 99.47 ± 7.85 ms, t = -5.89, *p* < 0.001), suggesting that MSI-type tumors have lower signals on T₂ mapping images.

ADC value: The ADC value in the MSI group was significantly higher than that in the MSS group (1.06 ± 0.18 vs. 0.91 ± 0.19 × 10⁻³ mm²/s, t = 4.78, *p* < 0.001), indicating that MSI-type tumors have less restricted water molecule diffusion.

There were no statistically significant differences in T₂ values and ADC values measured by different brands of MRI scanners (Philips Elition and Siemens Prisma) within the MSI group (T₂ value: 91.52 ± 6.21 vs. 91.38 ± 6.25 ms, *p* = 0.921; ADC value: 1.05 ± 0.15 vs. 1.04 ± 0.16 × 10⁻³ mm²/s, *p* = 0.876), indicating that scanner differences had no significant impact on the measurement results.

**Fig. 2:**
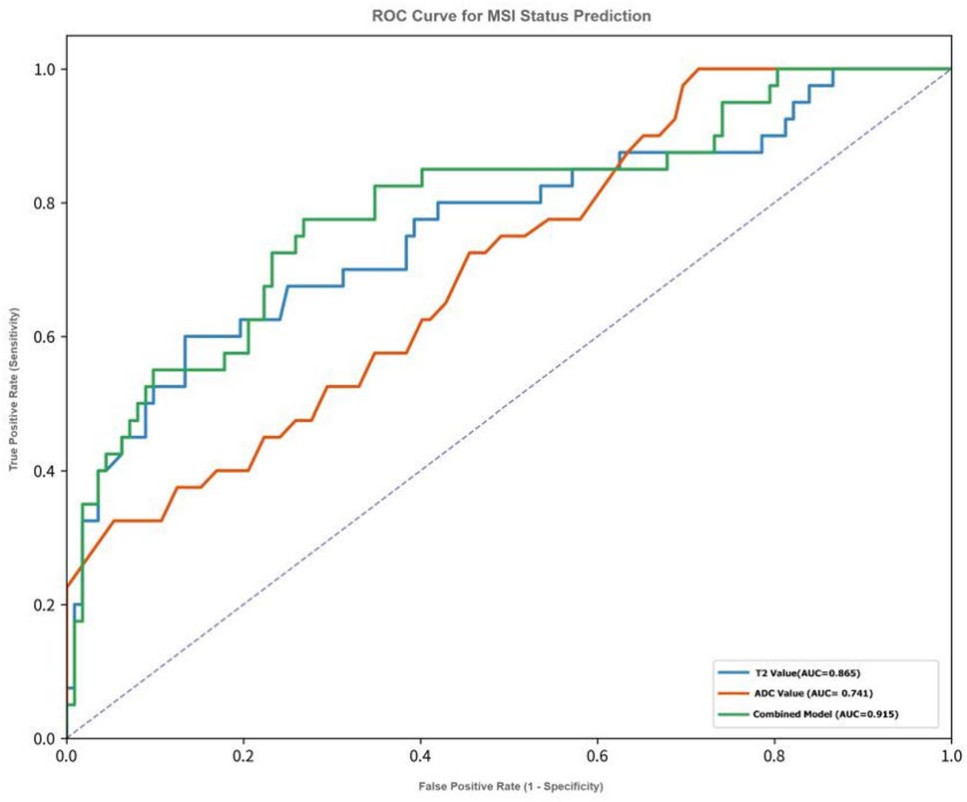
ROC curve for MSI status prediction, including reference line (AUC = 0.5), T2 value (AUC = 0.865), ADC value (AUC = 0.741), and combined model (AUC = 0.915, with 95% CI interval band marked)

ROC curve analysis (see Fig. 2) showed that single imaging parameters had predictive value for the MSI status of rectal cancer:

T₂ value: AUC = 0.865 (95% CI: 0.798–0.932), optimal cutoff value = 94.5 ms, Maximum Youden Index = 66.4%, corresponding sensitivity = 82.5%, specificity = 83.9%, positive predictive value (PPV) = 66.0%, negative predictive value (NPV) = 92.7%.

ADC value: AUC = 0.741 (95% CI: 0.652–0.830), optimal cutoff value = 0.97 × 10⁻³ mm²/s, Maximum Youden Index = 46.4%, sensitivity = 75.0%, specificity = 71.4%, PPV = 51.7%, NPV = 87.5%.

In comparison, the diagnostic efficacy of T₂ value was significantly superior to that of ADC value (Delong test: Z = 2.41, *p* = 0.016).

### Comparison of imaging parameters among different clinical stages

One-way ANOVA results (Table 2) showed:

**Table 2:** Diagnostic performance of the combined model across different clinical stages

Clinical Stage	Number of Cases (MSI/MSS)	AUC (95% CI)	Sensitivity (%)	Specificity (%)	PPV (%)	NPV (%)
Stage I	12/28	0.89 (0.81–0.97)	83.3	89.3	76.2	92.9
Stage II	18/50	0.92 (0.86–0.98)	82.2	90.0	77.8	92.3
Stage III	10/34	0.90 (0.82–0.98)	80.0	88.2	72.7	91.2

T₂ value: There was a statistically significant difference among different clinical stages (F = 3.48, *p* = 0.033). Specifically, the T₂ value in stage III (99.75 ± 6.90 ms) was significantly higher than that in stage I (96.72 ± 7.50 ms, *p* = 0.043), suggesting that the T₂ value tends to increase with tumor progression.

ADC value: There was no statistically significant difference among different clinical stages (F = 0.93, *p* = 0.401), indicating that the ADC value is less affected by tumor stage.

### Construction of multivariate logistic regression model

Taking “MSI status” as the dependent variable, a binary logistic regression model was constructed by including T₂ value, ADC value, and potential confounding factors (BMI, tumor location, image quality score) (combined with SMOTE oversampling to address class imbalance and L2 regularization to avoid overfitting). The results are shown in Table 3:

**Table 3:** Parameters of the multivariate logistic regression model (verified by bootstrap resampling)

Variable	β coefficient	Standard error (SE)	Wald χ² value	*p*-value	OR (95% CI)
Intercept	-13.15	3.38	15.22	< 0.001	-
T₂ value	0.09	0.03	7.31	0.006	1.09 (1.03–1.16)
ADC value	4.32	1.43	8.98	0.002	74.15 (8.53-643.21)
BMI	-0.05	0.03	2.81	0.094	0.95 (0.89–1.01)
Tumor location (middle vs. low)	0.13	0.36	0.13	0.719	1.14 (0.55–2.30)
Image quality (2 points vs. 1 point)	-0.22	0.39	0.32	0.573	0.80 (0.40–1.59)

**Fig. 3:**
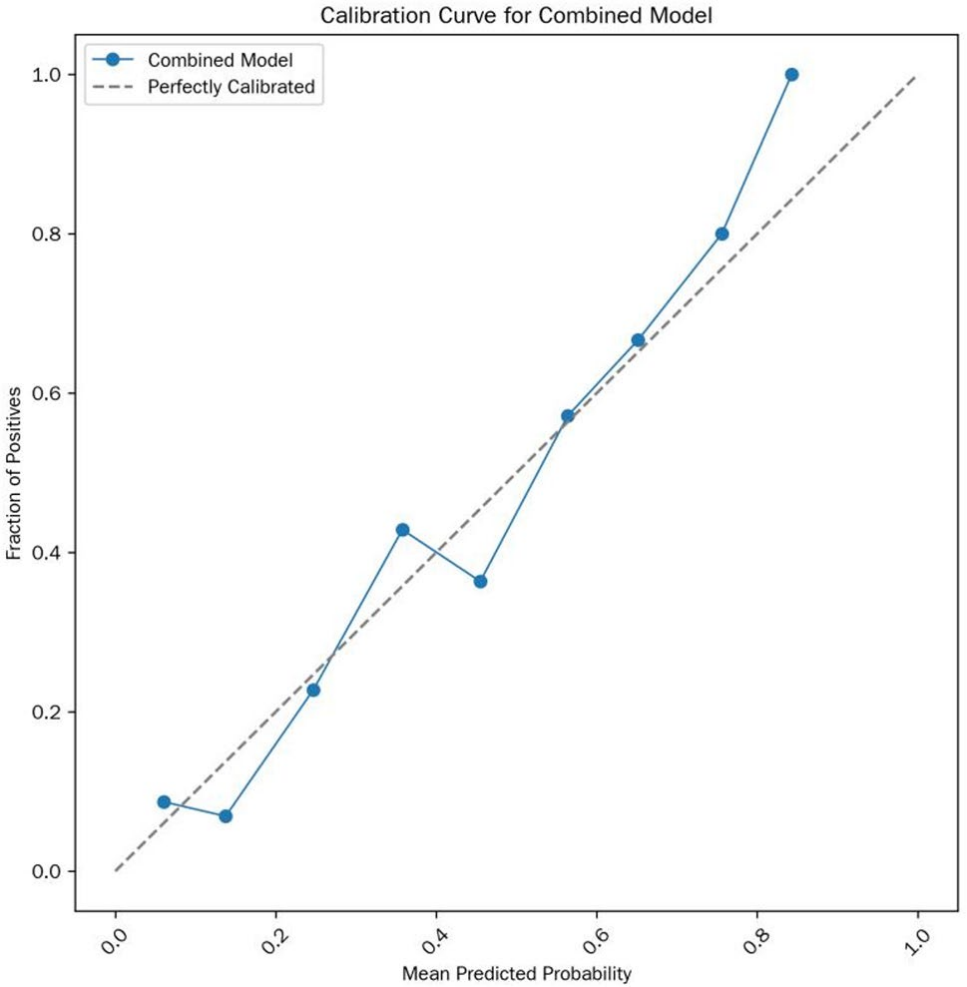
Calibration curve of the combined model; most points are close to the ideal calibration line; Brier score = 0.078; Hosmer-Lemeshow test *p* = 0.554

Model formula: Logit(P) =-13.15 + 0.09×T₂ value + 4.32×ADC value (where P is the predicted probability of MSI status). The calibration curve is shown in Fig. 3.

### Diagnostic efficacy of the combined model

The diagnostic performance of the combined prediction model based on T₂ value + ADC value was significantly superior to that of single parameters:

ROC curve (Fig. 2): AUC = 0.915 (95% CI: 0.865–0.965), Maximum Youden Index = 71.8%, sensitivity = 82.5%, specificity = 89.3%, PPV = 75.0%, NPV = 93.8%.

Comparison with single parameters: The AUC of the combined model was significantly higher than that of T₂ value (Z = 2.43, *p* = 0.015) and ADC value (Z = 4.05, *p* < 0.001).

Two methods were used to verify model stability:

10-fold cross-validation: Mean AUC = 0.906 (95% CI: 0.850–0.962), and the AUC fluctuation range across folds was < 5%.

Bootstrap resampling (1000 times): Mean AUC = 0.913 (95% CI: 0.865–0.961), indicating that the model maintained stable diagnostic performance across different sample subsets.

**Fig. 4:**
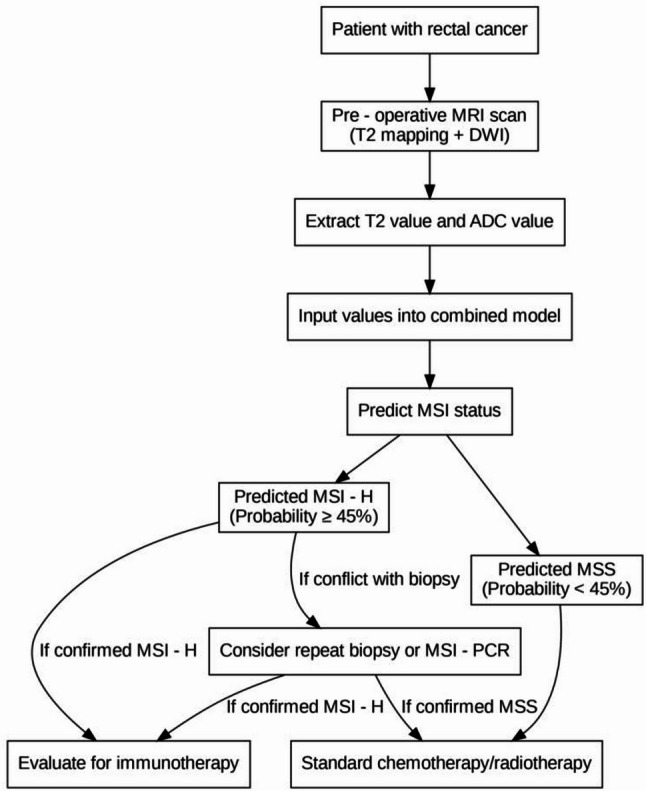
Clinical decision-making flowchart for MSI prediction. Note: Optimal cutoff probability = 45% (determined by the maximum Youden index); if there is a conflict between the model prediction and biopsy results, repeated MSI-PCR verification is recommended

The clinical decision-making flowchart for MSI prediction is shown in Fig. 4.

## Discussion

Microsatellite instability (MSI) status in rectal cancer is a key biomarker guiding postoperative prognostic evaluation and immunotherapy decision-making. However, the current invasive assessment relying on biopsy has limitations such as sampling bias (e.g., insufficient representativeness of local samples due to tumor heterogeneity) and reporting delay (3–5 days of turnaround time), which may not fully meet the clinical needs of preoperative precise stratification. By analyzing preoperative MRI data from 152 patients with pathologically confirmed rectal cancer, this study systematically verified the synergistic value of T₂ mapping quantitative parameters (T₂ value) and apparent diffusion coefficient (ADC value) in predicting MSI status for the first time. Its core findings not only provide new evidence for non-invasive assessment but also expand existing knowledge from the perspectives of imaging-pathology correlation and clinical adaptability.

### Pathological mechanisms and biological significance of imaging parameter differences

This study observed that the T₂ value in the MSI group was significantly lower than that in the microsatellite stability (MSS) group (92.18 ± 7.21 ms vs. 99.47 ± 7.85 ms), while the ADC value in the MSI group was significantly higher than that in the MSS group (1.06 ± 0.18 vs. 0.91 ± 0.19 × 10⁻³ mm²/s). These differences are unlikely to represent purely numerical variations and may instead reflect distinct tumor microenvironment characteristics associated with MSI-type rectal cancer. Previous pathological studies have reported that MSI-high (MSI-H)/deficient mismatch repair (dMMR) rectal cancers are typically characterized by increased tumor-infiltrating lymphocytes (TILs) and reduced stromal fibrosis [9, 10].

A reduction in stromal fibrosis may decrease the proportion of bound water within the tissue and increase the relative fraction of free water, thereby shortening the T₂ relaxation time and resulting in lower signal intensity on T₂ mapping [11]. In parallel, although TILs are cellular components, they have lower cytoplasm abundance and nuclear density compared with tumor cells, resulting in a weaker restrictive effect on water molecule diffusion. Additionally, reduced fibrosis further decreases the obstruction of extracellular matrix to diffusion, collectively leading to an increase in the ADC value in the MSI group [12]. It should be noted that this study has not yet conducted precise matching analysis between preoperative MRI and postoperative large tissue sections. The aforementioned mechanisms are still reasonable inferences based on literature. Future studies need to clarify the quantitative correlation between T₂ value, ADC value, TILs density, and stromal fibrosis degree through imaging-pathology correlation research to verify this hypothesis.

### Differences in diagnostic efficacy between single parameters and combined models: basis for clinical selection

Receiver operating characteristic (ROC) analysis showed that the area under the curve (AUC) of T₂ value alone for predicting MSI (0.865) was significantly better than that of ADC value (0.741), suggesting that T₂ mapping may provide a more robust single-parameter performance than diffusion-derived metrics in this cohort. This result is consistent with the conclusion of Zheng et al. [8], who reported the advantages of T2WI in evaluating the microstructure of rectal cancer. Notably, the present study extends these observations by employing T₂ mapping, which enables quantitative analysis rather than qualitative signal assessment.

Unlike conventional T₂-weighted imaging, which relies on visual interpretation of signal intensity and may be influenced by scanner settings and reader experience, T₂ mapping provides objective and reproducible measurements of tissue relaxation properties. This quantitative nature contributes to improved interobserver consistency, as reflected by the high intraclass correlation coefficient (ICC > 0.90) observed for both T₂ and ADC values in this study. Therefore, the favorable diagnostic performance of T₂ mapping observed here may be attributable not only to its discriminative ability but also to its methodological advantages in terms of stability and reproducibility.

Although the ADC value has relatively weak diagnostic efficacy when used alone, there was no significant difference in ADC value across different clinical stages (F = 0.93, *p* = 0.401), while the T₂ value showed a significant upward trend with the progression of clinical stages (Stage III: 99.75 ± 6.90 ms vs. Stage I: 96.72 ± 7.50 ms, *p* = 0.043). This characteristic suggests that the ADC value may be a stage-independent stable predictor for MSI, especially suitable for advanced-stage (Stage II/III) rectal cancer. At this stage, the increased degree of fibrosis in the tumor microenvironment may interfere with the judgment of T₂ value, while the ADC value is less affected by staging and can serve as a supplementary indicator. In contrast, the T₂ value has higher diagnostic value in early-stage (Stage I) rectal cancer, as it can more sensitively reflect the microenvironmental differences of early MSI tumors.

The combined model constructed based on the above characteristics (Logit(P) = -13.15 + 0.09×T₂ value + 4.32×ADC value) increased the AUC to 0.915. Compared with the T₂ value alone, the specificity (89.3%) and negative predictive value (93.8%) of the combined model increased by 5.4% and 1.1%, respectively. From a clinical perspective, higher specificity may reduce the risk of misclassifying microsatellite-stable tumors as MSI, thereby avoiding unnecessary consideration of immunotherapy in patients unlikely to benefit, while a high negative predictive value may help reliably exclude MSI status and provide supportive information for selecting conventional chemotherapy regimens. Compared with the previous MSI prediction model based on radiomics by Zhang et al. [7] (AUC = 0.88), the combined model in this study relies solely on quantitative parameters derived from routine MRI post-processing, without the need for complex feature extraction or machine-learning algorithms, which may facilitate broader clinical implementation, particularly in primary or resource-limited centers.

### Optimization value for the precision diagnosis and treatment workflow of rectal cancer

It should be emphasized that biopsy-based histopathological and molecular analyses remain the gold standard for the diagnosis of rectal cancer and determination of MSI status. The MRI-based approach proposed in this study is not intended to substitute histological assessment, but rather to provide complementary, non-invasive information that may support clinical decision-making, particularly in cases with equivocal biopsy results or limited tissue sampling.

In the current practice, high-resolution MRI is routinely used for preoperative evaluation of rectal cancer, including tumor staging (T/N staging) and circumferential resection margin assessment, whereas MSI status evaluation still relies primarily on invasive biopsy. By incorporating T₂ mapping and DWI sequences, both of which are routinely available MRI sequences without additional scanning time, quantitative assessment of T₂ and ADC values enables the potential integration of MSI-related information into standard preoperative MRI examinations, allowing a more comprehensive imaging-based evaluation.

The integrated imaging strategy may offer several practical advantages. First, it may help reduce the need for repeated or additional biopsy procedures in selected clinical scenarios, such as deeply located tumors or cases complicated by intestinal stenosis, particularly when initial biopsy results are inconclusive. Second, quantitative MRI parameters reflect the overall microenvironmental characteristics of the tumor rather than local biopsy tissues, which may help mitigate sampling bias associated with intratumoral heterogeneity, thereby providing supportive evidence alongside histopathological findings. Third, MSI-related imaging information can be obtained concurrently with routine staging results, which may assist multidisciplinary discussions and early treatment planning while awaiting definitive pathological confirmation.

In addition, subgroup analysis across different clinical stages in this study showed that the combined model maintained high diagnostic efficacy in Stage I–III rectal cancer (AUC = 0.89–0.92), with optimal performance observed in Stage II rectal cancer (AUC = 0.92). Considering that patients with Stage II MSI-H rectal cancer have significantly lower postoperative recurrence risk than MSS patients [1] and that immunotherapy has clear benefits for MSI-H patients [3], this model may provide supportive information for postoperative risk stratification. Importantly, such imaging findings should be interpreted in conjunction with pathological results and other clinical factors, rather than serving as a standalone basis for treatment decisions. In this context, the proposed model may contribute to individualized treatment planning by complementing histological assessment, particularly in cases with ambiguous or limited biopsy information.

### Study limitations and future research directions

Several limitations should be acknowledged in this study. First, this study was a single-center retrospective study, which may limit the generalizability of the findings. Although the sample size of the MSI group (*n* = 40) was consistent with the overall incidence of MSI-H in rectal cancer (3%–5%) and met the requirements of a priori sample size calculation, the relatively small number of patients in certain subgroups (e.g., Stage III disease) may reduce statistical power for stratified analyses. In addition, although two different 3.0-T MRI systems were used, imaging protocols were harmonized within a single institution, and therefore may not fully capture inter-institutional variability across different scanners, vendors, and field strengths. As a result, the diagnostic performance observed in this study may represent an upper estimate under standardized conditions. Future multicenter prospective studies incorporating diverse populations and imaging platforms (including both 1.5-T and 3.0-T systems) are warranted to validate the external robustness of the proposed model.

Second, this study focused exclusively on T₂ value and ADC values, and did not include other potentially informative imaging parameters (such as dynamic contrast-enhanced MRI [DCE-MRI], e.g., Ktrans and Ve values) or diffusion kurtosis imaging [13, 14]. While this simplified approach was intentionally chosen to enhance clinical feasibility and reproducibility, it may have limited the ability to capture additional aspects of tumor angiogenesis and microstructural heterogeneity. Consequently, the diagnostic efficacy of the model might be further improved by incorporating multiparametric MRI features in future investigations.

Third, direct quantitative correlation between imaging parameters and histopathological features, such as tumor-infiltrating lymphocytes or stromal fibrosis, was not performed. This limitation is inherent to the retrospective design and the lack of spatially matched whole-mount pathological sections. Although the biological interpretations proposed in this study are supported by existing literature [14], the absence of imaging–pathology fusion analysis precludes definitive mechanistic validation. Prospective studies integrating spatially matched imaging and histological analyses are needed to provide direct biological evidence for the observed imaging differences.

Finally, although optimal cutoff values were derived to facilitate statistical evaluation, these thresholds should not be interpreted as absolute diagnostic boundaries in clinical practice. In patients with borderline imaging values, integration with histopathological findings and multidisciplinary discussion remains essential. Future studies may explore probabilistic or continuous risk models, as well as the integration of imaging biomarkers with circulating tumor DNA–based MSI assessment [15, 16], to further enhance clinical decision support, particularly in patients for whom adequate tumor tissue is unavailable.

## Conclusion

This study confirms for the first time that T₂ mapping combined with ADC value can serve as a reliable method for non-invasive assessment of MSI status in rectal cancer. The combined model not only has better diagnostic efficacy than single parameters but also maintains stable performance across different clinical stages, demonstrating the potential for one-stop integration into routine MRI evaluation. Despite limitations such as single-center design and small sample size, this study provides a new paradigm for imaging-based evaluation of MSI in rectal cancer. Through simple and reproducible quantitative parameters, preoperative prediction of MSI status can be achieved, providing a basis for precision stratified treatment (e.g., screening for immunotherapy candidates). In the future, through multi-center validation, technical standardization, and multimodal integration, this method is expected to become an important part of routine preoperative evaluation of rectal cancer, promoting the advancement of rectal cancer diagnosis and treatment toward “imaging-guided individualized treatment”. 

## Data Availability

The data that support the findings of this study are available from the corresponding author upon reasonable request.

## Abbreviations

ANOVA: Analysis of Variance
AUC: Area Under the Curve
BMI: Body Mass Index
CI: Confidence Interval
dMMR: Deficient Mismatch Repair
MMR: Mismatch Repair
MRI: Magnetic Resonance Imaging
MSI: Microsatellite Instability
MSI-H: Microsatellite Instability-High
MSI-L: Microsatellite Instability-Low
MSS: Microsatellite Stability
ROI: Region of Interest
ROC: Receiver Operating Characteristic
